# Flexural Behavior of Fire-Damaged Prefabricated RC Hollow Slabs Strengthened with CFRP versus TRM

**DOI:** 10.3390/ma13112556

**Published:** 2020-06-04

**Authors:** Zheng-Ang Sui, Kun Dong, Jitong Jiang, Shutong Yang, Kexu Hu

**Affiliations:** 1 Department of Civil Engineering, Ocean University of China, Qingdao 266100, China; 21170911086@stu.ouc.edu.cn (Z.-A.S.); jtjiang@ouc.edu.cn (J.J.); yangshutong@ouc.edu.cn (S.Y.); 2 Collage of Civil Engineering, Tongji University, Shanghai 200092, China; kexuhu@tongji.edu.cn

**Keywords:** textile reinforced mortar, fiber reinforced polymer, strengthening, fire damage, pre-stressed hollow slabs

## Abstract

In this paper, carbon fiber reinforced polymer (CFRP) and textile reinforced mortar (TRM) strengthening techniques were proposed to retrofit and strengthen fire-damaged prefabricated concrete hollow slabs. A total of six slabs, from an actual multi-story masonry building, were tested to investigate the flexural performance of reinforced concrete (RC) hollow slabs strengthened with TRM and CFRP. The investigated parameters included the strengthening method (CFRP versus TRM), the number of CFRP layers, and with or without fire exposure. One unstrengthened slab and one TRM strengthened slab served as the control specimens without fire exposure. The remaining four slabs were first exposed to ISO-834 standard fire for 1 h, and then three of them were strengthened with CFRP or TRM. Through the four-point bending tests at ambient temperature, the failure modes, load and deformation response were recorded and discussed. Both CFRP and TRM strengthening methods can significantly increase the cracking load and peak load of the fire-damaged hollow slabs, as well as the stiffness in the early stage. The prefabricated hollow slabs strengthened by CFRP have better performance in the ultimate bearing capacity, but the ductility reduced with the increase of CFRP layers. Meanwhile, the TRM strengthening technique is a suitable method for the performance improvement of fire-damaged hollow slabs, in terms of not only the load capacity, especially the cracking load, but also the flexural stiffness and deformation capacity.

## 1. Introduction

Several severe destructions could lead up to performance degeneration of reinforced concrete (RC) structures. Fire hazard, a major sort of these destructions, brings a large number of economic losses and casualties every year. Due to the material property degradation and concrete cracking, even concrete spalling under high temperature conditions, the bearing and deformation capacity of RC members will be greatly degraded after fire exposure [1,2,3]. However, survey data indicated that few RC structures collapsed in or after a fire, and most structures had their residual bearing capacity to keep working [4,5,6,7]. Furthermore, most capacities of fire-damaged structures could be restored to a normal level by proper strengthening methods [8,9]. Therefore, it is essential and urgent to develop efficient strengthening solutions for fire-damaged structures and investigate the structural behavior after that.

In the past two decades, the fiber reinforced polymer (FRP) strengthening method is widely used in the reinforcement of RC structures [10,11,12]. Compared with the traditional methods, such as the cross-sectional enlargement using concrete or mortar overlays [13] and the sticking steel plates method [14,15], the FRP strengthening method has the advantage of lightweight, high strength and convenient construction for fire-damaged RC members [16,17,18,19,20,21]. Although the feasibility and efficiency of the FRP strengthening method at ambient temperatures have been verified, this method also has its non-negligible drawback, the poor heat resistance of epoxy resins and adhesives used in FRP reinforcement [22,23]. At elevated or high temperatures, the mechanical performance of FRP strengthened members would degrade greatly because of the adhesive softening and thermal stresses at the bond interface, inducing by the thermal incompatibility of FRP and concrete [24,25]. These drawbacks will make the strengthened members more dangerous at another fire or high temperature exposure, which restricts the application of FRP strengthening method in the field of concrete structure reinforcement. Therefore, FRP strengthening method was usually used in the actual projects under ambient temperature environment.

Currently, spraying textile reinforced mortar (TRM) on concrete surfaces became another one of the new reinforcement methods [26,27]. TRM, also known as fabric-reinforced cementitious matrix (FRCM), is composed of fiber fabric mesh and high strength polymer cement mortar. In contrast to the cross-sectional enlargement method, fiber fabric mesh was used to replace reinforced steel bars, and polymer cement mortar was instead of concrete or mortar overlays. With its material composition, TRM material has better compatibility with substrate concrete [28,29], better durability [30], and better resistance to high temperatures [31]. With these advantages, TRM has been used as external reinforcement for RC members. Most of the existing researches about TRM strengthened members were carried out at ambient temperatures, for improving the flexural load capacity of RC beams and slabs [32,33], the biaxial bending performance of RC columns [34], and the seismic resistance of masonry-infilled RC frames [35]. Based on the existing research studies as mentioned above, TRM is an attractive candidate for the retrofitting and strengthening of fire-damaged RC members.

Although both carbon fiber reinforced polymer (CFRP) and TRM methods were suitable for strengthening the concrete members, only few experimental researches were conducted to investigate the different strengthening effects between the two methods, on the seismic resistance [36] and high temperature resistance [37,38,39]. From the existing literature survey, the subject of TRM vs. FRP in the strengthening of fire-damaged prefabricated hollow RC slabs has not covered and needs to be studied for the promotion of CFRP and TRM reinforcement technology.

This paper reports a series of static tests on fire-damaged prefabricated hollow slabs, respectively strengthened with CFRP or TRM, in which the effects of different methods and different numbers of CFRP layers were investigated. After that, a calculation method of the bearing capacity of TRM strengthening fire-damaged hollow RC slabs was presented.

## 2. Specimens and Materials

### 2.1. Specimens Design

**Figure 1:**
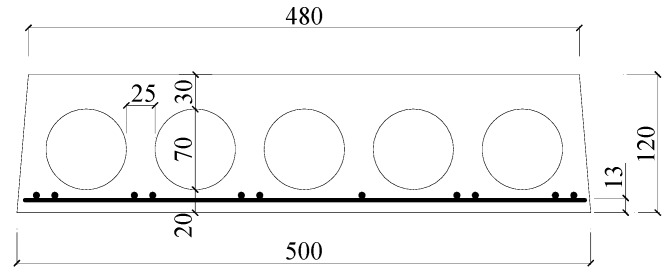
Details of the hollow slab (all dimensions in mm).

To evaluate the performance of TRM versus FRP in the flexural capacity of fire-damaged RC prefabricated hollow slabs at high temperatures is the main purpose of the current study. A total of six existing prefabricated RC hollow slabs were exposed to fire, strengthened by TRM versus FRP, and tested under a four-point bending load in this study. These hollow slabs came from an actual multi-story masonry building and were fabricated in 1993, with the same dimensions of 120 mm thickness × 500 mm width × 3900 mm length. As shown in Figure 1, the hollow slabs were produced in the same dimensions and reinforcement layout.

Five holes, with a diameter of 70 mm and spaced at 25 mm, was designed at the cross-section for each RC hollow slab, while positioned 30 mm and 20 mm away from the top and bottom of the slab, respectively. Each slab has 11 longitudinal prestressed steel rebars with a nominal diameter of 4 mm, while the same rebars spaced at 200 mm were used as the transverse reinforcement. The clear concrete cover to the longitudinal rebars was 13 mm.

In these tests, two unstrengthened slabs served as the control specimens, S0 without fire exposure and SF0 with one hour of ISO 834 standard fire exposure. Depending on the different strengthening methods, the other four specimens were divided into two series, one strengthened by CFRP and the other by TRM. SFC1 and SFC2 were exposed to fire for 1 hour prior to strengthening, and then flexural strengthened respectively with one layer and two layers of CFRP sheets. Two layers of TRM were applied to the fire-damaged specimens SFT2, as well as the undamaged specimens ST2. The details of all the specimens are shown in Table 1.

### 2.2. Properties of Materials

**Figure 2:**
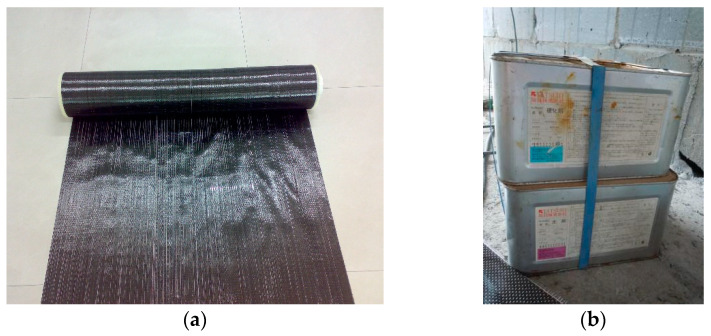
Materials in carbon fiber reinforced polymer (CFRP) strengthening method. (**a**) CFRP sheets; and (**b**) Epoxy resin.

Since the hollow slabs were from the existing structure, the material properties of concrete and steel rebars were measured by laboratory tests. The average concrete strength was 40.1 MPa, measured by the rebound method according to Chinese code JGJT 23 [40]. To measure the strength of prestressed steel rebars, six rebar samples were taken out from the slabs, three of which were tested before fire exposure, while the other three samples after fire exposure. Taking the strength corresponding to 0.2% residual plastic strain as nominal yield strength, the yield strength of steel bar is 500 MPa before fire exposure, and 400 MPa after fire exposure. The ultimate strength is 660 MPa, regardless of whether fire exposure or not. CFRP sheets HT300 as the external reinforcement in the tests were supplied by Japanese Tatsuhi Co., Ltd., (Tokyo, Japan) as well as the matched epoxy adhesive TH-RESIN (Figure 2).

The measured properties of CFRP and epoxy adhesive are presented in Table 2. It is worth noting that the tensile strength of CFRP sheets was 3379 MPa, five times than the ultimate strength of prestressed steel rebars. The shear bond strength measured by the steel-steel tensile shear test was 24.5 MPa, which can strongly guarantee CFRP materials and RC hollow slab to work jointly.

**Figure 3:**
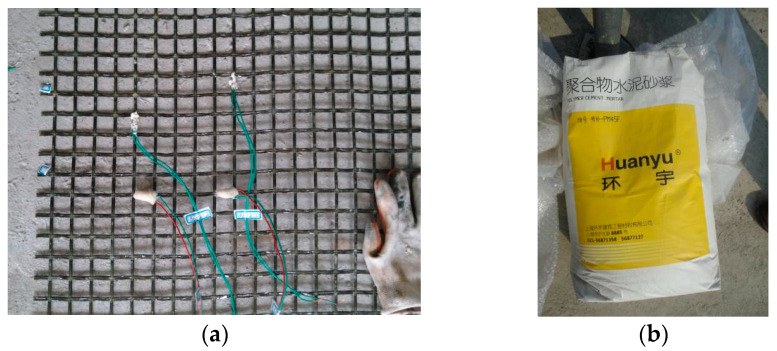
Materials in textile reinforced mortar (TRM) strengthening method. (**a**) Basalt fabric mesh; and (**b**) Polymer cement mortar.

The TRM strengthening layer consists of fiber fabric mesh and polymer cement mortar, as illustrated in Figure 3. The polymer cement mortar is a type of cement-based mortar modified by polymer material to improve adhesion, flowability, and strength. In this test, polymer cement mortar PM45F was used as the reinforcement matrix, which was provided by Shanghai Huanyu Construction Engineering Materials Company. According to the standard JGJ/T 7 [41], three mortar cubes with dimensions of 70.7 mm × 70.7 mm × 70.7 mm were made to measure the strength and elastic modulus. The average compressive strength of the polymer cement mortar after 28-day curing was 43.5 MPa. The properties of the polymer cement mortar, given by the test results and the manufacture’s datasheets, are described in Table 3.

In practical engineering, the fiber fabric mesh is an excellent building material for enhancing the anti-seepage and crack resistance of cement concrete. In the TRM strengthening technique, like the steel rebars, fiber fabric mesh was employed as the tensile member in the strengthening layer to bear the load. The fiber fabric mesh BGA25 used in the test was supplied by Dongguan Guangdong Russia & Gold Basalt Fiber Co., Ltd. As can be seen in Figure 3a, the fabric mesh was two-way weaved by basalt fabrics, with a mesh space of 25 mm in both longitudinal and transverse directions.

The properties of basalt fiber fabric mesh are summarized in Table 4, in which all the values were provided by the manufacturer. It should be noted that the tensile strengths were different in the two directions, due to the different weaving types for fabricating the basalt fabric mesh. Through measured with a Vernier caliper, every single roving was about 3.8 mm wide and 1.2 mm thick. The average tensile strength of single fiber roving in the longitudinal direction was measured as 442.2 MPa. It is equivalent to 80.7 kN/m for the fabric mesh, because there are 40 fiber rovings within a 1 m width. Compared the given data with the measured longitudinal strength in the longitudinal direction, it can be found that the tensile strength for single roving was higher than that for the entire fabric mesh. This mainly due to the uneven loading for measuring the basalt fabric mesh.

## 3. Fire Exposure Test

### 3.1. Fire Test Setup

The fire tests were carried out in Fire Resistance Horizontal Test Furnace of Tongji University (Shanghai, China). The test furnace consists of a fire chamber, control system and data acquisition system. The fire chamber had dimensions of 4.5 m long, 3.0 m wide and 2.2 m deep. The control system can automatically control the furnace temperature and pressure following a given curve, and the data acquisition system can automatically collect the temperature and displacement data of specimens.

**Figure 4:**
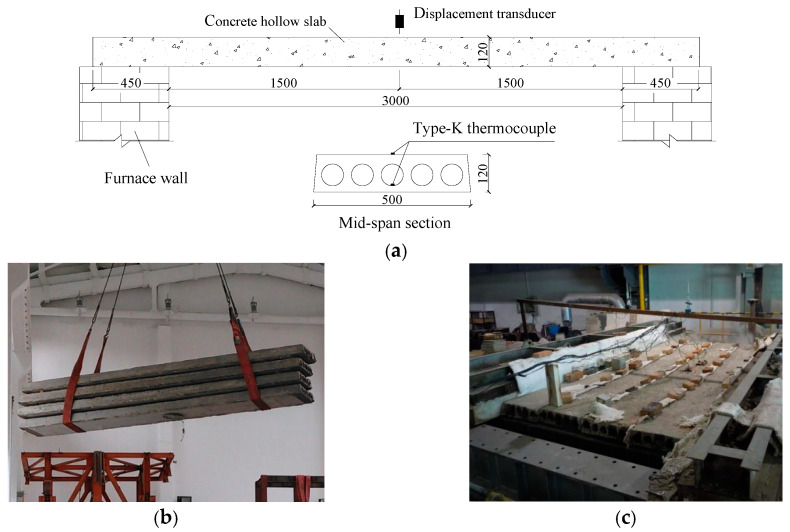
Test details for fire exposure (unit: mm). (**a**) Instrumentations of the specimens (all dimensions in mm); (**b**) Specimens before fire; (**c**) Specimens in fire test.

Four specimens, including SF0, SFC1–2, and SFT2, were placed one by one along the width direction of the furnace chamber and then exposed to standard fire. As shown in Figure 4, all the four slabs were tested at one time, and the clear span of the slabs in the furnace was 3000 mm. The two ends of the slabs were simply supported on the furnace wall in an unloaded condition, and then rock wool blankets were used to fill and cover the gaps between two adjacent hollow slabs and between the slabs and the furnace covers to form enclosed space and reduce the heat loss. It should be noted that these specimens have not been strengthened yet, and there is an 8 mm thickness plastering layer attached to the bottom of the slabs.

Ten internal thermocouples monitor the furnace temperature during a fire test, and a total of four type-K thermocouples were placed in the mid-span section of two slabs to record the temperature of core holes and the top surface of slabs. Besides, mid-span deflections were measured by displacement transducers.

Considering that the maximum fire resistance of concrete slabs was generally required for 1.5 h of standard ISO834 fire exposure, so the heat time in this test was set as 60 min. After 1 h of fire exposure, the fire test was over, and the prefabricated hollow slabs were naturally cooled to room temperature before being removed out of the furnace.

### 3.2. Fire Response

**Figure 5:**
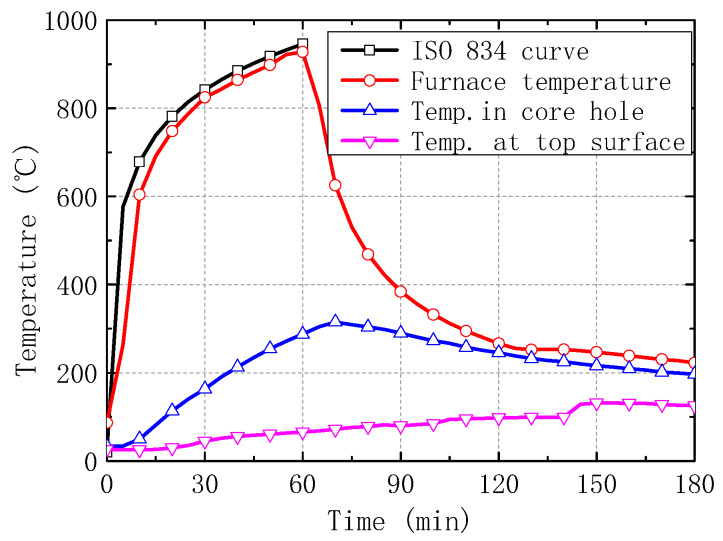
Time-temperature curves for slabs.

**Figure 6:**
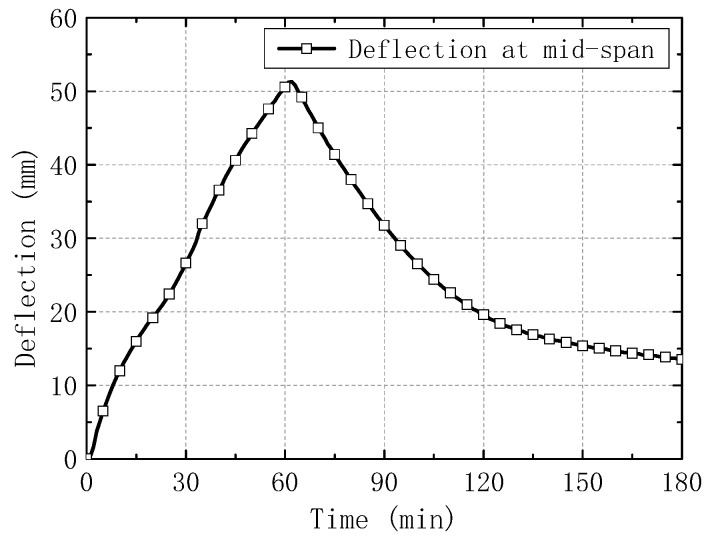
Time-deflection curve for slabs.

**Figure 7:**
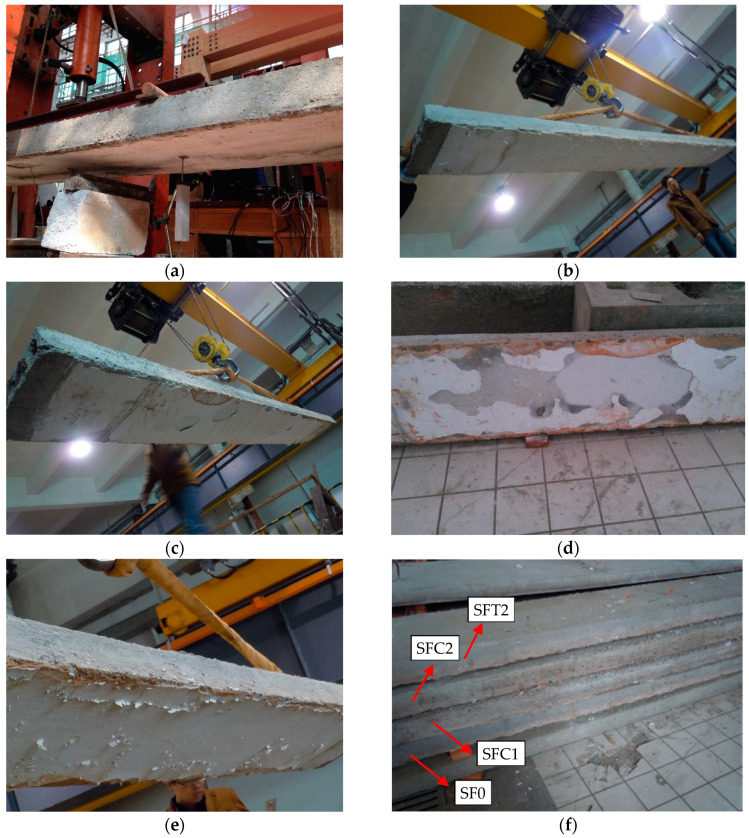
Observations after the fire test. (**a**) Non-fire exposed S0; (**b**) Fire-damaged SF0; (**c**) Fire-damaged SFC1; (**d**) Fire-damaged SFC2; (**e**) Fire-damaged SFT2; (**f**) The side surface.

The measured average temperatures of furnace chamber and hollow slabs, the mid-span deflection, and the post-fire photos of slabs are respectively shown in Figure 5, Figure 6 and Figure 7.

In the fire test, the furnace temperature was following the time-temperature curve of ISO 834 standard fire. The maximum temperature automatically recorded in the chamber was 927.3 °C. During the fire and cooled process, the average temperature at the top of slabs was only 70 °C at 60 min, and up to 130 °C at 150 min (Figure 5), due to the heat transition from the bottom to the top of slabs in the enclosed furnace. The average temperature in the holes reached 290 °C at 60 min of fire exposure and then elevated to a maximum value of 320 °C at 10 min after the fire stopped. From that it can be deduced the max temperature of prestressed rebars was more than 320 °C during the test, which may lead to some strength degradation.

As also shown in Figure 6, the deflection of the slabs increased linearly and reached 50 mm when the test ended at 60 min. The deflection change can be mainly contributed to the uneven expansion between the upper and lower section of concrete slabs. After that, the deflection reduced to 13.5 mm at 180 min, and 5 mm after cooled for one day to room temperature.

After being cooled down naturally for one day, the temperature of the whole hollow slabs had dropped to room temperature, and the specimens were removed from the test furnace. As a reference, Figure 7a shows a picture of the bottom surface of non-fire exposed specimen S0, from which the intact plastering layer with no damage was observed. Then it could be observed from Figure 7b–e that few mortars of the plastering layer fell off, and some temperature cracks appeared on the surface of the plastering layer. On the side surface of the slabs, the concrete about a quarter height from the bottom changed color to pink, along with some small cracks on it in the mid-span region, as shown in Figure 7f. Due to the thermal isolation effect of the 8 mm thick plaster layer and the poor thermal conductivity of concrete, no concrete burst and no substantial changes occurred on the upper section of the slabs.

## 4. Four-Point Bending Test

### 4.1. Strengthening Procedure

After the fire test, three fire-damaged hollow slabs and one non-fire exposed slab were strengthened by the CFRP or TRM reinforcement method. Two 1.8 m-high masonry walls spaced at 3.2 m were built to support both ends of unstrengthened slabs, serving as an assistance in reinforcement construction. As listed in Table 1, the flexural capacity of each slab was enhanced by only one strengthening method.

**Figure 8:**
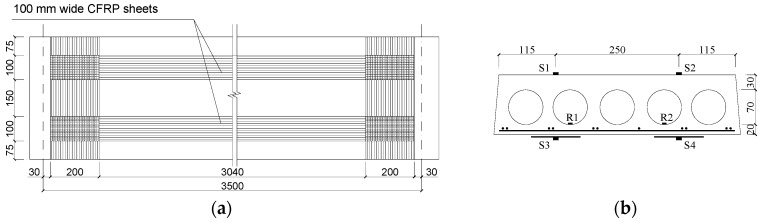
Schematic diagram of SFC1and SFC2 (unit: mm). (**a**) The bottom surface of slabs; (**b**) The cross-section at mid-span.

**Figure 9:**
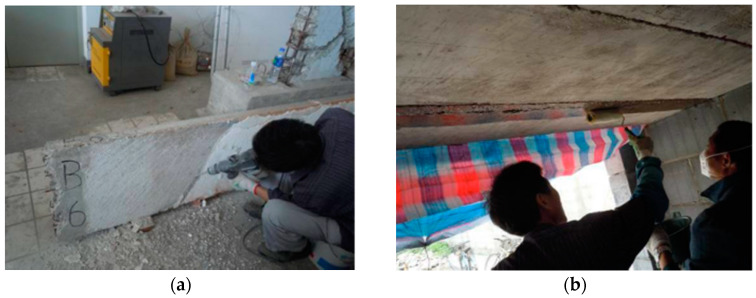

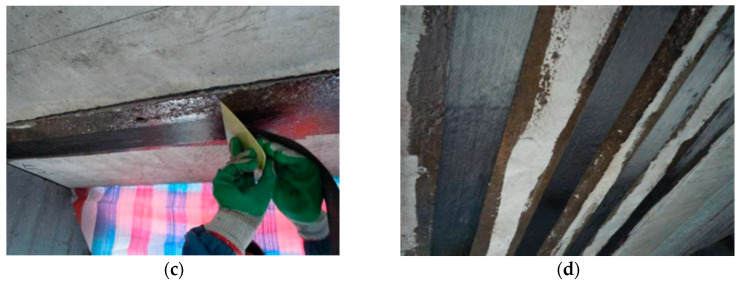
CFRP strengthening procedure of the specimens SFC1and SFC2 (unit: mm). (**a**) Surface treatment; (**b**) Apply the primer resin; (**c**) Paste the CFRP sheets; (**d**) Cure in an open environment.

For FRP retrofitted specimens SFC1 and SFC2, Figure 8 shows the details of the strengthening design and the arrangement of gauges. The strengthening procedure composed of four steps. First, make concrete surface treatment. After the plastering layer under the slabs was removed, as shown in Figure 9a, the concrete surface was polished and roughened by an angle grinder. The slurry layer, where the CFRP was applied, was removed up to expose to the coarse aggregate (i.e., about 3–5 mm thick). Second, apply the primer resin (Figure 9b). The two-component epoxy resin was used as the primer adhesive, which can permeate into the concrete surface in an hour and enhance the interfacial bonding property. Next, paste the CFRP sheets when the primer was touch dry (Figure 9c). Two pieces of 100 mm wide CFRP sheets were parallelly placed at the bottom surface of the slabs with a center-to-center space of 250 mm and a length of 3440 mm. After that, a piece of transverse CFRP sheet with a width of 200 mm was used at each end to improve the anchorage effect. Before and after placing each CFRP sheet, one layer of epoxy resin (TH-RESIN) was applied using a plastic roller, to achieve a better bond between the carbon fibers and the surrounding matrix. The above steps were repeated for specimen SFC2 when another layer of CFRP sheet was used. Finally, cure in an open environment (Figure 9d). After curing for about one day, the resin changed from fluid to solid with lower bond strength, and most of the ultimate strength can be achieved after curing for seven days.

**Figure 10:**
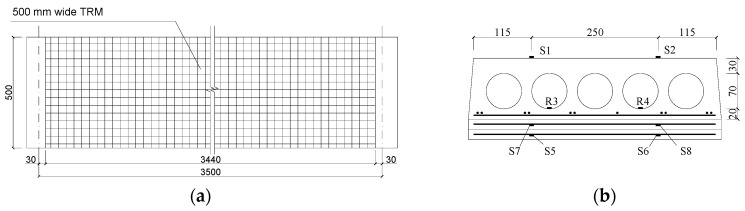
Schematic diagram of SFT2 and ST2 (unit: mm). (**a**) The bottom surface of slabs; (**b**) The cross-section at mid-span.

**Figure 11:**
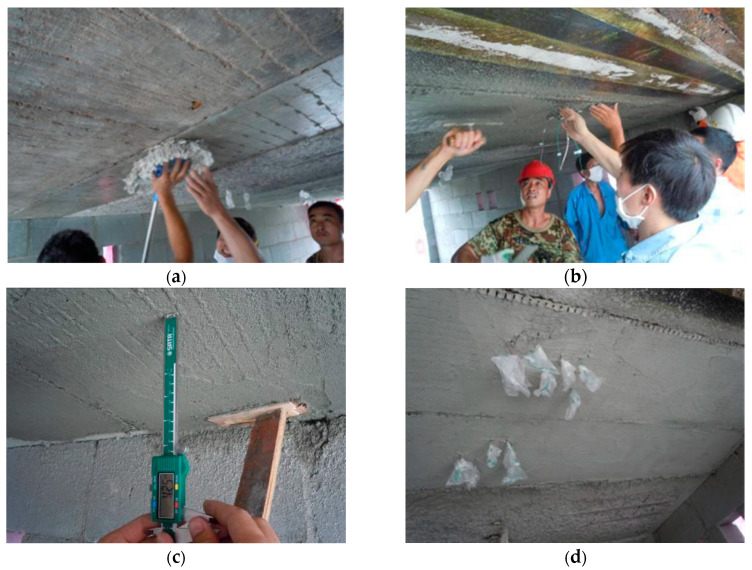
TRM strengthening procedure of specimens SFT2 and ST2 (unit: mm) (**a**) Surface treatment; (**b**) Apply the fiber mesh; (**c**) Thickness measurement; (**d**) Curing naturally.

For TRM retrofitted specimens SFT2 and ST2, Figure 10 shows the details of the strengthening design and the arrangement of gauges. The TRM strengthening steps were similar with the FRP retrofitted specimens. In the step of surface treatment (Figure 11a), some transverse grooves with a depth of about 2 mm were set on the soffit of the slabs using a churn drill, to improve the bond between the concrete and the external polymer cement mortar. After that, a thin layer of about 1.5 mm thick polymer cement mortar was sprayed onto the moistened concrete substrate as the adhesion agent.

Before the installation of the TRM strengthening system, the basalt fabric mesh was impregnated with TH-resin for 2 days in advance to enhance the interaction behavior of basalt fibers. Two strain gauges were attached to the fabric mesh in the mid-span. First, a layer of polymer cement mortar PM45F with a thickness of 8 mm was sprayed directly onto the surface of the adhesion agent, and one layer of impregnated basalt fabric mesh was immediately applied and pressed horizontally and evenly into the polymer cement mortar (Figure 11b). Then, the second mortar layer of 8 mm thickness was sprayed. The same steps were used for the outer TRM layer, consisting of the second basalt fabric mesh and the third layer of polymer cement mortar. The thickness of each layer was measured by a Vernier caliper to meet the design requirement (Figure 11c). After the entire retrofit procedure was finished, the TRM strengthened specimens were naturally cured for 28 days, as shown in Figure 11d.

### 4.2. Load and Measure Arrangement

The flexural bending tests were conducted on all the six specimens, including two unstrengthened hollow slabs and four strengthened slabs (see more details in Table 1). The two-point loading system was adapted in the test, as shown in Figure 4. A distributing girder was used to distribute the load from a servo-hydraulic actuator with a maximum load capacity of 100 kN to the two loading points on the concrete hollow slab. The distance between the loading points was 1167 mm, and the clear span of the specimens was 3500 mm.

**Figure 12:**
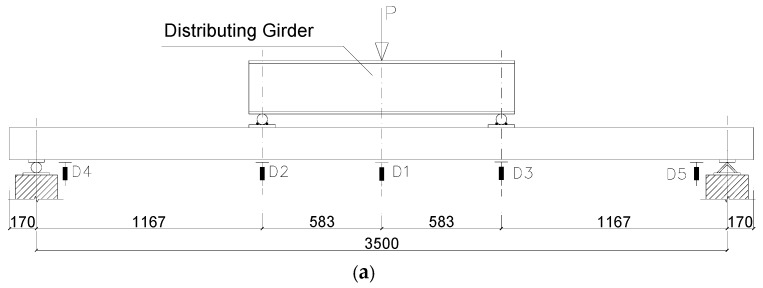

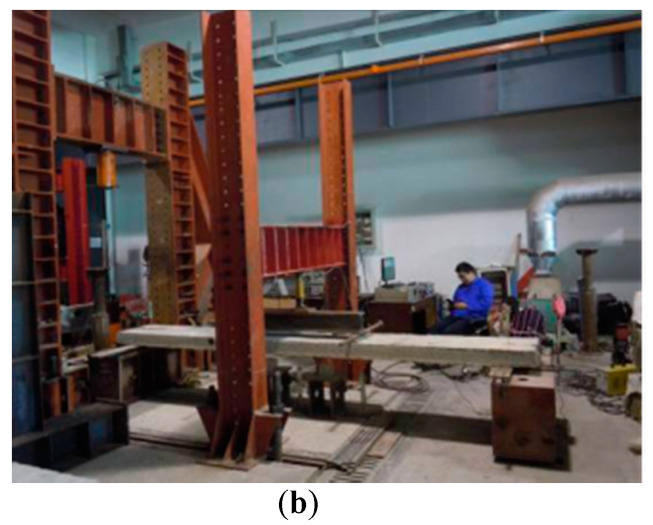
The set-up of the four-point bending test. (**a**) The details of test set-up (all dimensions in mm); (**b**) General view for the bending test.

For each specimen, five displacement sensors, D1–D5, were used to measure and record the deflection of the specimens at mid-span, one-third span, and support location, as shown in Figure 12a. Additionally, the strain measurement of the top surface of concrete, CFRP sheet and basalt fabric at mid-span was made by eight strain gauges, S1–S8, as shown in Figure 8b and Figure 10b. It should be noted that only the CFRP strain at the outer layer was measured, while the TRM strain measurement was set for each layer. The crack load, peak load and crack development of the specimens were investigated. A photograph of the four-point test on CFRP versus TRM strengthened fire-damaged hollow slabs is shown in Figure 12b.

### 4.3. Failure Modes and Crack Distribution

**Figure 13:**
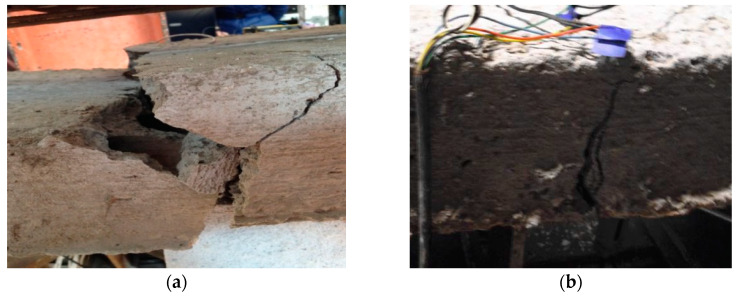

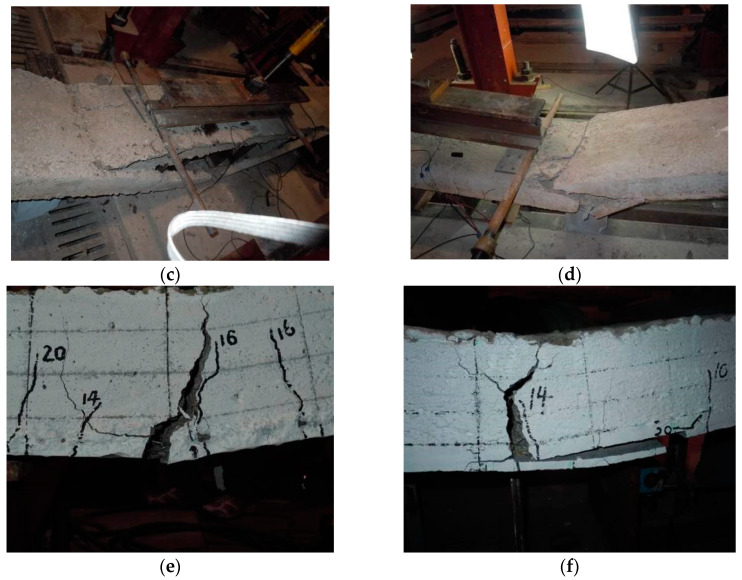
Failure mode of the specimens. (**a**) S0; (**b**) SF0; (**c**) SFC1; (**d**) SFC2; (**e**) SFT2; (**f**) ST2.

Figure 13 shows the failure modes of specimens after the flexural bending test. During the test, the main crack on specimen S0 appeared in the middle zone of the span, and finally the hollow slab broke into two parts along the mid-span crack (Figure 13a), which was the bending failure due to the rupture of prestressed steel rebars. For specimen SF0, an arched crack appeared around the left support in the early stage. However, the reason caused to the bending failure was also the rupture of prestressed steel rebars at mid-span (Figure 13b).

Specimen SFC1 was strengthened with one layer of the CFRP sheet after fire exposure. The main crack in the bending test happened at the section, 800 mm away from the support. The crack developed with an approximately 30 degrees inclination. Finally, the failure mode of SFC1 was a shear failure, due to the premature falling of local concrete on the side face (Figure 13c). Specimen SFC2 was strengthened with two layers of CFRP after fire exposure. In the test, the diagonal cracks in the shear zone were rapidly developed and penetrated through the entire section. At last, the CFRP sheets peeled off from the slab soffit, and the shear fracture of the hollow slab was observed around the right loading point (Figure 13d).

Both specimens SFT2 and ST2 strengthened with two layers of TRM were bending failure. For specimen SFT2, fracture failure of fabric mesh and pre-stressed steel bar occurred in the constant moment region around the right loading point (Figure 13e). Due to the mistake in the on-site lifting process, specimen ST2 had a transverse crack on the top surface near the mid-span before TRM retrofitted treatment. Under static loading, the initial crack in the compressive zone closed, which has little influence before the peak load reached. However, with the increasing of the load, the bending cracking occurred and developed from the bottom to the top part of cross-section, finally it happened to bend failure at the section where the initial crack located. As shown in Figure 13f, the pre-stressed steel bar ruptured and the TRM layer peeled off from the original concrete slab at the main crack zone.

**Figure 14:**
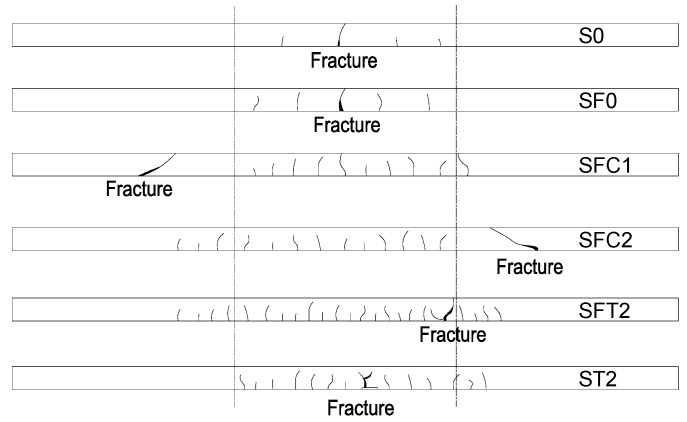
Cracks distribution in each specimen.

The final crack distribution at the side face was also observed and plotted in Figure 14 for each specimen. The dotted lines in figure marked the position of two loading points, between which the section moment kept constant.

As shown in Figure 14, most cracks occurred in the pure bending section, and the average spacing was measured as 248 mm for S0, 233 mm for SF0, 130 mm for SFC1, 117 mm for SFC2, 78 mm for SFT2 and 97 mm for ST2. It is illustrated that both CFRP and TRM reinforcement can make concrete cracks more uniformly distributed and more compact than unstrengthened specimens S0 and SF0. Compared with CFRP strengthened specimens, TRM strengthened specimens had better work performance, because of the more compact and short cracks when the failure load was reached. Expect for specimens SFC1 and SFC2, all the other specimens behaved bending failure, and the failure cracks developed along the vertical direction within the constant moment region. Furthermore, both the two TRM strengthened specimens have short peeling cracks between polymer cement mortar layer and concrete substrate, with a length of only about 5 cm. Consequently, it can be concluded that the TRM strengthening method had a satisfactory bonding performance between the strengthened layer and the original structure.

### 4.4. Load Response

Table 5 shows the recorded results during the bending test, including the cracking load, peak load and failure deflection at mid-span, as well as the failure mode. Under the test conditions in this paper, the cracking load of specimens SFC1, SFC2 and SFT2 increased respectively by 19%, 20% and 58%, and the peak load of specimens SFC1, SFC2 and SFT2 increased by 132%, 189% and 93%, respectively. After being strengthened, the failure deflection of hollow slabs had a more than a hundred percent increase for both methods. Therefore, both CFRP and TRM reinforcement methods can succeed to restore even significantly increased the load and deformation capacity of the prefabricated hollow slabs after fire exposure.

#### 4.4.1. Effect of Fire-Damage

**Figure 15:**
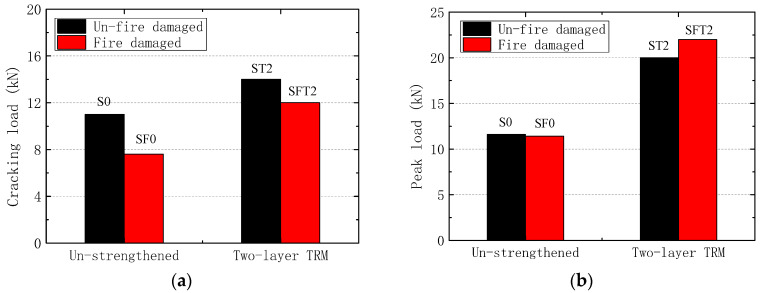
The comparison between undamaged and fire-damaged specimens. (**a**) Cracking load; (**b**) Peak load.

From Figure 15a, the cracking load of the fire-damaged specimens is generally less than that of the specimens without fire exposure, regardless of strengthened or unstrengthened slabs. The cracking resistance of the specimens mainly depended on the prestress level of the steel rebars and the tensile strength of the concrete. During the fire test, the bottom concrete was subjected to a high temperature of more than 320 °C, so the tensile strength was weakened even after cooled down to normal temperature. In addition, the prestressing loss was also caused by the fire-damage of the concrete around the prestressed rebars, which decreased the bond strength between concrete and steel rebars. However, after retrofitted and strengthened with TRM, no significant effect of the fire damage was found on the flexural bearing capacity of the prestressed hollow slabs. The failure load of fire-damaged specimen SFT2 and undamaged specimen ST2 was 22 kN and 20 kN (Figure 15b), respectively. The similar peak loads were contributed to the little strength change of concrete and prestressed steel rebars, and the initial crack on the top surface of ST2. During the fire test, the concrete of the upper section always kept a lower temperature with a maximum temperature of 130 °C at the top surface, so the compressive strength of the concrete was not damaged. Moreover, the material test showed that the tensile ultimate strength of the steel rebar was still 660 MPa, due to the thermal protection by the concrete cover and plaster layer. It also should be mentioned that the little lower peak load of ST2 might result from the initial crack on the top surface.

#### 4.4.2. Effect of Strengthening Method and Layers

**Figure 16:**
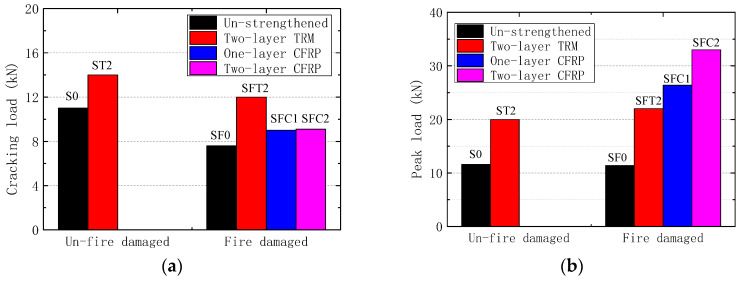
The comparison of specimens strengthened with different methods. (**a**) Cracking load; (**b**) Peak load.

As can be seen from Figure 16, the cracking load of SFC1 and SFC2 were not different, but the peak load of SFC2 increased up to 57% than that of SFC1. On the other hand, the ultimate deflection of SFC2 was lower by 22.3% than the specimen SF1 but higher by 90.9% than the unstrengthened fire-damaged specimen SF0. The results in Figure 16 and Table 5 show that with the increase of CFRP layers, the ultimate bearing capacity of the strengthened specimens increased, and the ductility reduced with the failure deflection from 135 mm to 105 mm.

In comparison, the CFRP strengthened specimens SFC1 and SFC2 had the higher peak loads, respectively by 20% and 50% than TRM strengthened fire-damaged slab SFT2. Two main reasons resulting in the variation of load capacity are the differences in the reinforcement amount and stress transfer mechanism. According to the different reinforcement amounts, the ultimate tensile load of reinforcements is about 80.7 kN for SFT2, 112.9 kN for SFC1, and 225.7 kN for SFC2. Furthermore, the reinforcements in CFRP method have a better continuous strain compatibility with the concrete slab, which could make the interface stress transfer more uniform and effective. In the case of TRM method, the cracking of the mortar matrix results in the tension stiffening effect inside the TRM layer, and the basalt fabric near the cracks will be premature tensile rupture due to the non-uniform stress. On the other hand, the cracking load of specimen SFT2 is higher than that of CFRP strengthened slabs, which indicated that TRM reinforcement can effectively improve the stiffness and capacity of the fire-damaged slabs in the elastic stage. The test results also show that the crack resistance and bearing capacity were improved greatly for TRM strengthened hollow slabs ST2 and SFT2, compared to that of unstrengthened slabs S0 and SF0.

The brittle inorganic matrix is cracked after a certain load level. In this case, a tension stiffening mechanism is activated inside the TRM until failure accompanied by the detachment.

### 4.5. Deformation Response

**Figure 17:**
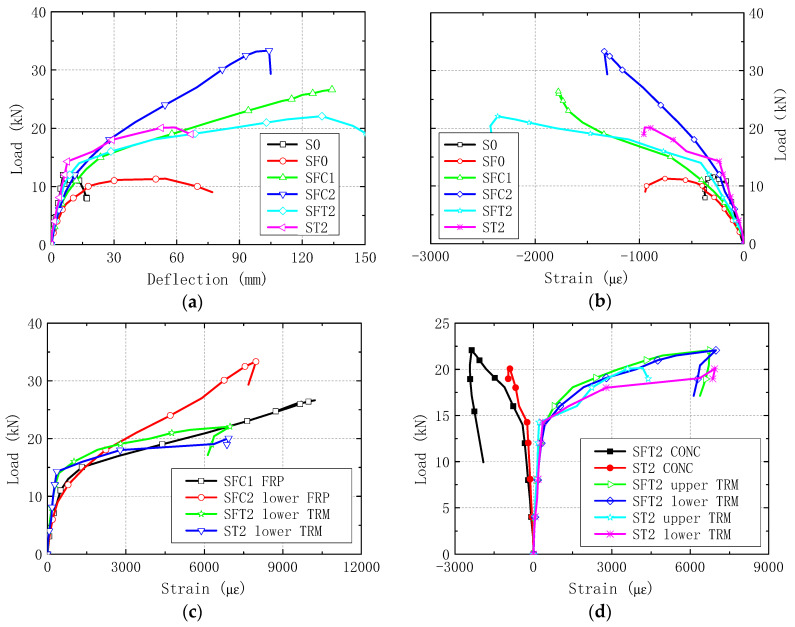
Load-deformation curves. (**a**) Load-deflection; (**b**) Load-concrete strain; (**c**) Load-CFRP/TRM strain; (**d**) Load-strain in SFT2 and ST2.

The load-deformation curves of all slabs tested in this study are presented in Figure 17. It should be noted that the residual deformation from fire tests was not considered here.

Figure 17a depicts the load-deflection curves for all the six specimens. Although having the same peak load, the stiffness of specimen SF0 after cracking is always less than that of the non-fire specimen S0. It is indicated that the elastic modulus of concrete decreased under the effect of high temperature. After strengthened with CFRP or TRM, the stiffness, bearing capacity, and ductility of the fire-damaged specimens (SFC1, SFC1, and SFT2) were all improved obviously. Compared to specimens SFC1 and SFC2, the specimen SFT2 exhibited a higher cracking load with a smaller cracking deflection, and a lower peak load with a larger failure deflection. The reason is that the increase of section height by TRM method enhanced the flexural stiffness of hollow slabs, which is larger than the CFRP strengthened slabs before concrete cracks occurred. In the following stage of bending test, the integral rigidity of TRM strengthened slab reduced greatly with the growth of cracks.

It can be seen from Figure 17b that in the elastic stage, the slope of strain growth for non-fire exposed specimens were larger than the fire-damaged specimens, which reflects again the influence of fire. However, after the strengthening treatment, the concrete compressive strain of the hollow slabs can continue to grow in a higher load lever until shear or bending failure. These changes have demonstrated that the CFRP and TRM flexural reinforcement can effectively improve the strength utilization ratio of concrete in the compression zone.

For specimen SFC1 and SFC2, the CFRP strain growth is almost the same in the early of the bending test (Figure 17c). After the load exceeded 15 kN, the strain growth of SFC2 postponed behind that of specimen SFC1, which indicates that the bearing capacity of the specimens increases with the increase of CFRP layers. Compared SFC2 with SFT2 in Figure 17c, the basalt fabric strain of SFT2 has an obvious delayed increase in the early stage of loading, while increased much rapid than the strain of SFC2. It indicates that the initial stiffness of TRM strengthened slabs was larger than the CFRP strengthened slabs, and the later stiffness weakened because of the development of flexural cracks.

As can be seen in Figure 17d, the concrete strain of ST2 postponed behind that of SFT2 obviously, which may attribute to the stiffness change of initial concrete slab after fire exposure. The strain curves of the upper and lower TRM layers are synchronous, which indicated that the basalt fabric mesh at the upper and lower layers could work well together.

## 5. Conclusions

The static bending tests were conducted to investigate the flexural performance of the fire-damaged prefabricated concrete hollow slabs strengthened with CFRP or TRM layers. This paper presented the test results including failure modes, crack distribution, load response, and deformation response. Based on these results and discussions, the following conclusions can be drawn.

(a) Both the CFRP and TRM strengthening methods can significantly increase the cracking load and peak load of the fire-damaged prefabricated hollow slab, as well as the stiffness and deformation capacity. Compared to the unstrengthened fire damaged slab, the average cracking load increased by 19%, 20% and 58% respectively for one-layer CFRP, two-layer CFRP and TRM strengthening method, while the increase of peak load was 132%, 189% and 93% respectively. After strengthened, the failure deflection of hollow slabs had a more than a hundred percent increase for both methods.
(b) Compared to the CFRP strengthened hollow slabs, the fire-damaged slabs strengthened with TRM exhibited a higher cracking load with a smaller cracking deflection, and a lower peak load with a larger failure deflection. The reason is that the integral flexural stiffness of the TRM strengthened hollow slab was larger than the CFRP strengthened slabs before concrete cracks appeared, and then reduced greatly due to the rapid growth of concrete cracks.
(c) The cracking load of the fire-damaged specimens was generally less than that of the non-fire exposed specimens, regardless of strengthened or unstrengthened slabs, which can be attributed to the cracking of lower concrete and the yield strength degradation of steel rebars after fire exposure. However, comparing the results of the two TRM strengthened slabs, no significant effect of 60 min fire exposure was found on the peak loads of the hollow slabs, which indicated that the TRM strengthening method could restore most flexural capacity of fire-damaged hollow slabs.

## Figures and Tables

**Table 1:** Strengthening details of all the test specimens.

Specimens	Heating Period (min)	Strengthening Method	Dimensions of Strengthening Layers (mm)	
			Width	Total Thickness
S0	—	—	—	—
SF0	60	—	—	—
SFC1	60	One-layer CFRP	100 + 100	0.167
SFC2	60	Two-layer CFRP	100 + 100	0.334
SFT2	60	Two-layer TRM	500	24
ST2	—	Two-layer TRM	500	24

**Table 2:** Properties of CFRP sheets and epoxy adhesive.

Property	Tensile Strength (MPa)	Elastic Modulus (GPa)	Rupture Strain (%)	Bond Strength (MPa)
HT300	3379	242	1.7	—
TH-RESIN	48	2.68	1.65	24.5

**Table 3:** Properties of polymer cement mortar

Mortar Type	PM45F
Maximum aggregate particle size (mm)	0–2
Elasticity modulus (MPa)	22,322 (tested)
Compressive strength (MPa)	43.5 (tested)
Tensile bond strength (MPa)	1.2

**Table 4:** Properties of basalt fabric mesh.

Mesh Type	BGA25
Density (g/cm^2^)	350
Elasticity modulus (GPa)	89
Longitudinal tensile strength (kN/m)	50
Transverse tensile strength (kN/m)	40
Rupture strain (%)	3.1

**Table 5:** Cracking loads and failure loads.

Specimen	Cracking Load (kN)	Peak Load (kN)	Failure Deflection (mm)	Failure Mode
S0	11.0	11.6	17.4	BF-S
SF0	7.6	11.4	55	BF-S
SFC1	9.0	26.4	135	SF
SFC2	9.1	33.0	105	SF
SFT2	12.0	22.0	130	BF-F
ST2	14.0	20.0	60	BF-F

BF-S: Bend Failure with Steel Rupture. SF: Shear Failure. BF-F: Bend Failure with Fabrics Rupture

## References

[1] Julia W., Robert K. (2020). Assessing concrete strength in fire-damaged structures. Constr. Build. Mater..

[2] Kodur V.K.R., Dwaikat M.M.S., Dwaikat M.B. (2008). High-temperature properties of concrete for fire resistance modeling of structures. ACI Mater. J..

[3] Arioz O. (2007). Effects of elevated temperatures on properties of concrete. Fire Saf. J..

[4] Gao W.Y. (2017). Fire resistance of RC beams under design fire exposure. Mag. Concr. Res..

[5] Raut N.K., Kodur V.K.R. (2011). Response of high-strength concrete columns under design fire exposure. J. Struct. Eng..

[6] Chen Y.H., Chang Y.F., Yao G.C., Sheu M.S. (2009). Experimental research on post-fire behavior of reinforced concrete columns. Fire Saf. J..

[7] Huang Z. (2010). The behavior of reinforced concrete slabs in fire. Fire Saf. J..

[8] Zhou J., Wang L. (2019). Repair of fire-damaged reinforced concrete members with axial load: A review. Sustainability.

[9] Ma W.X., Yin C.X., Zhou J., Lu W. (2019). Repair of fire-damaged reinforced concrete flexural members: A review. Sustainability.

[10] (2008). ACI Committee 440.2R-08. Guide for the Design and Construction of Externally Bonded FRP Systems for Strengthening Concrete Structures.

[11] Esfahani M.R., Kianoush M.R., Tajari A.R. (2007). Flexural behavior of reinforced concrete beams strengthened by CFRP sheets. Eng. Struct. J..

[12] Dalfre G.M., Barros J.A.O. (2013). NSM technique to increase the load carrying capacity of continuous RC slabs. Eng. Struct. J..

[13] Thanoon W.A., Jaafar M.S., Kadir M.R.A., Noorzaei J. (2005). Repair and structural performance of initially cracked RC slabs. Constr. Build. Mater..

[14] Lu W., Ray K.L.S. (2014). Repair of fire-exposed preloaded rectangular concrete columns by post compressed steel plates. J. Struct. Eng..

[15] Jiang C.J., Lu Z.D., Li L.Z. (2017). Shear performance of fire-damaged reinforced concrete beams repaired by a bolted side-plating technique. J. Struct. Eng..

[16] Haddad R.H., Shannag M.J., Moh’d A. (2008). Repair of heat-damaged RC shallow beams using advanced composites. Mater. Struct..

[17] Haddad R.H., Al-Mekhlfy N., Ashteyat A.M. (2011). Repair of heat-damaged reinforced concrete slabs using fibrous composite materials. Constr. Build. Mater..

[18] Al-Kamaki Y.S.S., Al-Mahaidi R., Bennetts I. (2015). Experimental and numerical study of the behavior of heat-damaged RC circular columns confined with CFRP fabric. Compos. Struct..

[19] Gherdaoui M., Guenfoud M., Madi R. (2018). Punching behavior of strengthened and repaired RC slabs with CFRP. Constr. Build. Mater..

[20] Cao N.T., Pansuk W., Torres L. (2015). Flexural behavior of fire-damaged reinforced concrete slabs repaired with near-surfaced mounted (NSM) carbon fiber reinforced polymer (CFRP) rods. J. Adv. Concr. Technol..

[21] Lenwari A., Rungamornrat J., Woonprasert S. (2016). Axial compression behavior of fire-damaged concrete cylinders confined with CFRP sheets. J. Compos. Constr..

[22] Dong K., Hu K.X. (2016). Development of bond strength model for CFRP to concrete joints at high temperatures. Compos. B Eng..

[23] Dai J.G., Gao W.Y., Teng J.G. (2013). Bond-slip model for FRP laminates externally bonded to concrete at elevated temperature. J. Compos. Constr..

[24] Dong K., Hu K.X., Gao W.Y. (2016). Fire behavior of full-scale CFRP-strengthened rc beams protected with different insulation systems. J. Asian Archit. Build..

[25] Gao W.Y., Dai J.G., Teng J.G. (2018). Three-level fire resistance design of FRP strengthened RC beams. J. Compos. Constr..

[26] Schladitz F., Frenzel M., Ehlig D., Curbach M. (2012). Bending load capacity of reinforced concrete slabs strengthened with textile reinforced concrete. Eng. Struct..

[27] Kouris L.A.S., Triantafillou T.C. (2018). State-of-the-art on strengthening of masonry structures with textile reinforced mortar (TRM). Constr. Build. Mater..

[28] D’Ambrisi A., Feo L., Focacci F. (2013). Experimental analysis on bond between PBO-FRCM strengthening materials and concrete. Compos. B Eng..

[29] Raoof S.M., Koutas L.N., Bournas D.A. (2016). Bond between textile-reinforced mortar (TRM) and concrete substrates: Experimental investigation. Compos. B Eng..

[30] Donnini J. (2019). Durability of glass FRCM systems: Effects of different environments on mechanical properties. Compos. B Eng..

[31] Donnini J., Basalo F., Corinaldesi V., Lancioni G., Nanni A. (2017). Fabric-reinforced cementitious matrix behavior at high-temperature: Experimental and numerical results. Composites.

[32] Ebead U., Shrestha K.C., Afzal M.S., El Refai A., Nanni A. (2016). Effectiveness of fabric-reinforced cementitious matrix in strengthening reinforced concrete beams. J. Compos. Constr..

[33] Gao W.Y., Hu K.X., Dai J.G., Dong K., Yu K.Q., Fang L.J. (2018). Repair of fire-damaged RC slabs with basalt fabric-reinforced shotcrete. Constr. Build. Mater..

[34] Bournas D.A., Triantafillou T.C. (2013). Biaxial bending of reinforced concrete columns strengthened with externally applied reinforcement in combination with confinement. ACI Struct. J..

[35] Koutas L., Bousias S., Triantafillou T. (2014). Seismic strengthening of masonry-infilled RC frames with TRM: Experimental study. J. Compos. Constr..

[36] Bournas D.A., Triantafillou T.C., Zygouris K., Stavropoulos F. (2009). Textile-reinforced mortar versus FRP jacketing in seismic retrofitting of RC columns with continuous or lap-spliced deformed bars. J. Compos. Constr..

[37] Raoof S.M., Bournas D.A. (2017). TRM versus FRP in flexural strengthening of RC beams: Behavior at high temperatures. Constr. Build. Mater..

[38] Tetta Z.C., Bournas D.A. (2016). TRM vs FRP jacketing in shear strengthening of concrete members subjected to high temperatures. Compos. B Eng..

[39] Raoof S.M., Bournas D.A. (2017). Bond between TRM versus FRP composites and concrete at high temperatures. Compos. B Eng..

[40] (2011). Chinese Code JGJ/T 23. Technical Specification for Inspection of Concrete Compressive Strength by Rebound Method.

[41] (2009). Chinese Code JGJ/T 70. Standard for Test Method of Performance of Building Mortar.

